# 364 Evaluation of a continuously active surface disinfectant against Candidozyma auris

**DOI:** 10.1017/ash.2026.10700

**Published:** 2026-06-23

**Authors:** Cindy Muyna, Alejandro Medrano, Mario Noli Legarde III, Marceau Doze, Kelly Reveles, Catina Rodriguez, Jose Cadena Zuluaga

**Affiliations:** 1 South Texas Veterans Health Administration; 2 Veterans Affairs; 3 South Texas Veterans Health Care System; 4 South Texas Veterans Healthcare System; 5 The University of Texas at Austin; 6 University Of Texas Health Science Center at San Antonio, South Texas Vetrans Healthcare System

## Abstract

**Background:** The accurate monitoring of blood glucose levels is critical in managing diabetes, and the Accu-Chek glucometer is a widely used tool for this purpose. Ensuring the proper cleaning and maintenance of these devices is essential to prevent cross-contamination and ensure reliable readings. This study evaluated the effectiveness of the cleaning process of Accu-Chek glucometers and assessed the competency of healthcare staff in adhering to the recommended two-step cleaning procedure. **Methods:** A study was conducted within the Audie l. Murphy Memorial Veterans Hospital (ALMMVH), a part of the U.S. Department of Veterans Affairs that provides comprehensive healthcare services to veterans. The VA is one of the largest integrated healthcare systems in the United States, serving millions of veterans annually. A standardized two-step cleaning protocol was implemented, which included an initial wipe with a cleaning solution followed by a disinfectant wipe. Initial compliance was evaluated across 46 clinical areas involving 159 staff members. Staff training sessions were subsequently conducted to educate on the importance and methodology of the cleaning process. Competency assessments were performed: pre- and post-training to evaluate the staff’s understanding and execution of the cleaning procedure. Follow-up evaluations were conducted in 28 clinical areas with 91 staff members participating. Compliance was compared between the pre- and post-intervention periods using the chi-square test. **Results:** Initial assessments indicated a compliance rate of 55%, with notable variability in staff competency regarding the cleaning process. Post-training evaluations demonstrated significant improvement, with compliance rising to 88% for the two-step cleaning process. (p<0.001 compared to the pre-intervention period). The study observed a significant reduction in the number of dirty machines. Initially, out of 91 machines, 37 were classified as dirty. After implementation of interventions, the number of dirty machines decreased markedly to five out of 57. The comparison of clean machines between pre- and post- periods results in a p-value of <0.0001. The study highlights the effectiveness of training in improving standard operating procedures. **Conclusion:** The study underscores the critical role of proper training and adherence to cleaning protocols in ensuring the safety and accuracy of blood glucose monitoring. By reinforcing a standardized two-step cleaning process, healthcare facilities can significantly reduce the risk of cross-contamination may enhance reliability of glucometer readings. Ongoing education and competency evaluations are essential to sustain these improvements.

